# Memory NK Cell Features Exploitable in Anticancer Immunotherapy

**DOI:** 10.1155/2019/8795673

**Published:** 2019-02-06

**Authors:** Cristina Capuano, Chiara Pighi, Simone Battella, Angela Santoni, Gabriella Palmieri, Ricciarda Galandrini

**Affiliations:** ^1^Department of Experimental Medicine, Sapienza University of Rome, Rome 00161, Italy; ^2^Department of Molecular Medicine, Sapienza University of Rome, Rome 00161, Italy; ^3^Laboratorio Pasteur Italia Fondazione Cenci Bolognetti, Sapienza University of Rome, Rome 00161, Italy; ^4^IRCCS Neuromed, Pozzilli 86077, Italy

## Abstract

Besides their innate ability to rapidly produce effector cytokines and kill virus-infected or transformed cells, natural killer (NK) cells display a strong capability to adapt to environmental modifications and to differentiate into long-lived, hyperfunctional populations, dubbed memory or memory-like NK cells. Despite significant progress in the field of NK cell-based immunotherapies, some factors including their short life span and the occurrence of a tumor-dependent functional exhaustion have limited their clinical efficacy so that strategies aimed at overcoming these limitations represent one of the main current challenges in the field. In this scenario, the exploitation of NK cell memory may have a considerable potential. This article summarizes recent evidence in the literature on the peculiar features that render memory NK cells an attractive tool for antitumor immunotherapy, including their long-term survival and *in vivo* persistence, the resistance to tumor-dependent immunosuppressive microenvironment, the amplified functional responses to IgG-opsonized tumor cells, and *in vitro* expansion capability. Along with highlighting these issues, we speculate that memory NK cell-based adoptive immunotherapy settings would greatly take advantage from the combination with tumor-targeting therapeutic antibodies (mAbs), as a strategy to fully unleash their clinical efficacy.

## 1. Introduction

NK cells represent a pivotal player of innate antitumor immune responses. They can eradicate neoplastic cells by a targeted release of cytotoxic granules containing perforin and granzymes and/or death receptor-mediated killing [1]. Moreover, NK cells can signal to other immune cells by producing cytokines and chemokines, such as IFN-*γ*, TNF*α*, IL-6, GM-CSF, and CCL5 in response to target cells or cytokine stimulation [1, 2]. In particular, NK-derived IFN-*γ* stands as a well-recognized key immunoregulatory factor in the shaping of antitumor adaptive immune responses, by modulating dendritic cell (DC) and T cell responses [3–5]. Further, NK cell-mediated antibody-dependent cellular cytotoxicity (ADCC) is a main immune-dependent mechanism by which tumor-targeting therapeutic mAbs mediate tumor cell killing [6–8].

NK cell functional response to tumor cells encounter is triggered by a variety of activating receptors, some of which (e.g., NKG2D and DNAM-1) recognize stress-induced ligands expressed on malignantly transformed cells; additionally, NK cells are potently activated by CD16 or Fc*γ*RIIIa (low-affinity Fc receptor for IgG)-dependent recognition of IgG-opsonized targets. In contrast, individual NK cells express, in different combinations, several inhibitory receptors (e.g., CD94/NKG2A and killer immunoglobulin-like receptors (KIR)) that recognize MHC class I molecules. In addition to modulate functional responsiveness, NK cell inhibitory receptors are critical for promoting their education [9].

The perspective of NK cells as exquisitely innate effectors is challenged by the recent appreciation that NK cells can adapt their functional program in response to environmental factors, through the generation of long-lasting specialized NK cell populations with enhanced effector functions, named adaptive or memory NK cells [10–12].

The first demonstration of antigen-specific recall responses by NK cells was in the setting of hapten-induced contact hypersensitivity, where CXCR6^+^ liver-derived murine NK cells could mediate antigen-specific contact hypersensitivity independently from B and T cells [13, 14].

Doubtless, memory NK cell populations have been mostly extensively characterized in the setting of cytomegalovirus (CMV) infection. Murine CMV (MCMV) infection induces immunological memory independent of T and B cells [15, 16]. Protection in these models is mediated by Ly49H^+^ NK cells, which upon recognition of m157 viral antigen (Ly49H ligand) proliferate and persist in lymphoid and nonlymphoid organs. Upon reinfection, these memory NK cells undergo secondary expansion, rapidly degranulate, and release cytokines, leading to a protective immune response, and also provide protection to newborn mice challenged with MCMV, upon adoptive transfer [16].

Accordingly, human CMV (HCMV) deeply impacts on NK cell compartment; in such context, memory NK cells have been initially identified in healthy HCMV-seropositive individuals, mainly on the basis of high expression levels of CD94/NKG2C activating receptor and CD57 terminal differentiation marker [17–21]. Such NKG2C^+^ memory NK cells can constitute up to 70% of the total NK cell population and can persist at high frequency for years [22–25].

Similar to Ly49H in mice, NKG2C is a member of the C-type lectin superfamily and associates to the adaptor protein DAP12 [26]. NKG2C forms heterodimers with CD94 and binds to the nonclassical MHC class I molecule HLA-E bound to HLA-E-stabilizing peptides [27].

HCMV-associated NK cells exhibit a distinct surface receptor expression pattern, consisting of a reduced expression of NKG2A (the inhibitory receptor counterpart of NKG2C), NKp30, NKp46, and CD16, as well as an increased expression of ILT2 (LIR-1a) [11, 19]. Further, it has been reported that *ex vivo* memory NK cells display an oligoclonal KIR pattern, with a bias for self-specific members both in healthy donors and chronic hepatitis patients [18, 24].

These features, along with additional phenotypic hallmarks, including the preferential expression of the activating receptor CD2, together with the reduced expression of the inhibitory receptor Siglec-7 [28], collectively aid in the identification of this unique and discrete NK cell population.

A link between HCMV and memory NK cell expansion is supported by the finding of an increase in CD94/NKG2C^+^ NK cells following the HCMV reactivation or infection in patients receiving hematopoietic stem cell transplant [22, 23, 29–31] and strengthened by the recent identification of HCMV-encoded antigen UL40, as the HLA-E ligand that drives the *in vitro* expansion and differentiation of memory NKG2C^+^ NK cells [32]; however, a potential role of other receptors besides NKG2C in the recognition and response to HCMV infection and in the skewing of an identical cellular program has been proposed [33].

Seminal independent studies have identified an immune-receptor tyrosine-based activation motif (ITAM)-bearing Fc*ε*RI*γ* adaptor protein-deficient NK cell subset in HCMV-seropositive individuals, endowed with a specific epigenetic signature, mostly overlapping with the CD94/NKG2C^+^ population [19–21, 34, 35]. Fc*ε*RI*γ* chain deficiency became an important feature of memory NK cell population, together with the specific downregulation of PLZF and IKZF2 transcription factors, as well as the variable loss of the intracellular signaling molecules DAB2, SYK, and EAT-2.

Memory NK cells also display a distinctive genome-wide methylation profile that confers an overall epigenetic profile very similar to that of memory CD8^+^ T cells, thus providing a molecular basis for the adaptive features of these cells. In particular, the promoter regions of Fc*ε*RI*γ*, EAT-2, SYK, and PLZF genes are highly methylated in memory NK cells, compared to conventional NK cells (cNK). Likewise, the promoter regions of IL-12 and IL-18 receptor subunit genes, which are regulated by PLZF, are also highly methylated, accounting for a reduced ability to respond to bystander activation by these cytokines [12, 21].

Another major epigenetic hallmark of memory NKG2C^+^ NK cells is represented by a significant demethylation of the conserved noncoding sequence (CNS) 1 in the IFNG locus, which remains stable in progeny, similar to what occurs in memory Th1 cells [25]. This molecular signature provides a mechanism to explain the potent IFN-*γ* production in response to the stimulation through a selective recognition repertoire. Indeed, the engagement of NKG2C by HLA-E-expressing target cells potently activates memory NK cells and leads to polyfunctional responses characterized by degranulation as well as TNF*α* and IFN-*γ* production [18]. Further, memory NK cells can be efficiently stimulated by the cross-linking of CD16 through the recognition of Ab-coated virus-infected cells [19, 21, 33, 34].

Long-lived memory-like NK cells can also be generated in noninfectious or antigen-independent settings. Specifically, *in vitro* stimulation of mouse splenic NK cells with IL-12 and IL-18, prior to transfer into a naive host, generated a pool of cells with enhanced IFN-*γ* production in response to cytokines, activating receptor ligands or tumor targets [36, 37], without any enhanced cytotoxicity. Similar to murine memory-like NK cells, when human NK cells are preactivated with IL-12, IL-15, and IL-18 and subsequently rested for several days, they display an increased IFN-*γ* production upon restimulation with cytokines or target cells compared with control population and such enhanced activity is maintained following an extensive cell division [38, 39].

## 2. *In Vivo* Evidence of Memory NK Cell Antitumor Activity

Preclinical and clinical observations suggest that memory NK cell activities could be advantageous in tumor settings and may contribute to relapse protection, in the context of hematopoietic malignancies.

Several studies reported a longer relapse-free survival after allogeneic stem cell transplantation in acute myeloid leukemia (AML) or chronic myeloid leukemia (CML) patients experiencing HCMV reactivation [40–43]. Moreover, the expansion of NKG2C^+^CD57^+^ memory NK cells in leukemic patients that reactivated CMV following allo-hematopoietic stem cell transplant (HSCT) is associated with a significantly reduced rate of relapse [44], suggesting that the recognition of HLA-E^+^ leukemic blasts by memory NKG2C^+^ NK cells expanded in response to HCMV infection may have beneficial effect through the eradication of minimal residual disease.

Furthermore, consistent with the finding that murine cytokine-preactivated memory-like NK cells maintain enhanced antitumor activity after adoptive transfer [38], a single injection of human memory-like NK cells significantly reduced the leukemia burden and improved the overall survival compared with that of control NK cells, in a xenograft model of leukemia [45]. Similarly, an independent study also found effective control of melanoma growth by cytokine-preactivated human NK cells in a melanoma xenograft model in NOD scid gamma (NGS) mice. The enhanced antitumor effects mediated by memory-like NK cells might result from their augmented cytotoxicity, high IFN-*γ* production capacity, and persistence in large numbers in the host [46].

More importantly, a phase I clinical trial harnessing cytokine-induced memory-like NK cells was recently performed in patients with relapsed or refractory AML [45], which consisted in the adoptive transfer of donor-derived NK cells preactivated with IL-12, IL-15, and IL-18, following fludarabine/cyclophosphamide-mediated lymphodepletion. Tracking donor memory-like NK cells in recipients revealed that they underwent *in vivo* expansion. As expected, donor memory-like NK cells displayed higher frequencies of IFN-*γ*^+^ cells with respect to recipient NK cells when challenged *ex vivo* with K562 leukemia cells. Notably, five out of nine evaluable patients showed a clinical response, including four complete remissions, which compares favorably with previous studies utilizing purified NK cells without cytokine preactivation [47].

## 3. Unique Features of Memory NK Cells Exploitable in Cancer Immunotherapy

### 3.1. Long-Term Survival and *In Vivo* Persistence

A crucial aspect of memory or memory-like NK cells is a longer life span, with respect to conventional populations, along with the capability to mediate persistent responses.

While NK cells are considered short-lived effectors of innate immunity, with an estimated half-life of 14 days [48, 49], HCMV-induced CD94/NKG2C^+^ NK cells exhibit a persistence of several months in the absence of detectable viremia and were stably maintained at elevated frequency for years, in some healthy individuals [22, 23, 30]. Moreover, after the umbilical cord blood transplantation in patients with hematopoietic malignancies, CMV reactivation leads to a long-lasting increase of NKG2C^+^ NK cells [29, 31].

Recent studies, involving patients with either paroxysmal nocturnal hemoglobinuria or GATA2 deficiency, demonstrate that memory NK cells selectively persist in these patients in spite of a reduction of conventional NK cell populations, supporting an independent survival and self-renewal pathway for the homeostatic maintenance of CD56^+^ NK cells with an adaptive phenotype [50, 51].

Mechanistic studies demonstrated that human memory NK cells express higher levels of antiapoptotic Bcl-2 that marked an epigenetically unique population persisting for at least 35 months [21, 34]. The authors speculate that, analogously to long-lived and self-renewing memory T cells, the reduced expression of PLZF could support memory NK cell longevity.

**Figure 1:**
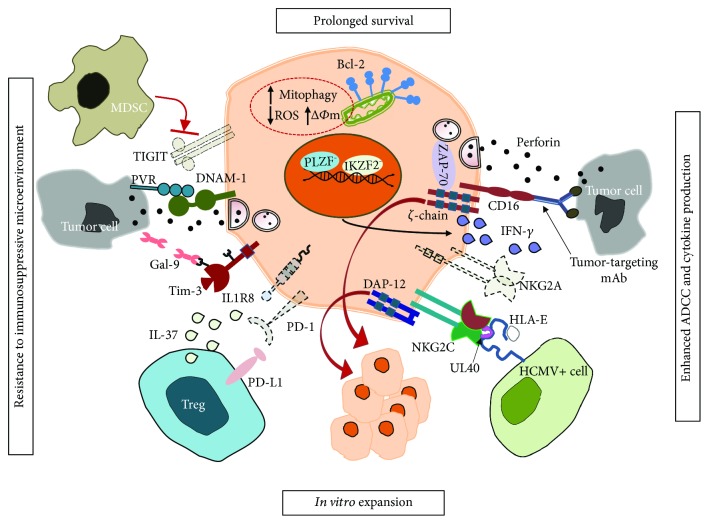
NKG2C^+^ memory NK cell features exploitable in cancer immunotherapy. The dotted lines indicate reduced receptor expression.

More recently, an isoform of AT-rich interaction domain 5 (ARID5B) transcription factor has been found selectively upregulated in memory NK cells and involved in promoting mitochondrial membrane potential, oxidative metabolism, survival, and IFN-*γ* production [52]. Collectively, such evidence provides molecular basis for memory NK cell longevity (Figure 1).

The MCMV infection model gave important insights on memory NK cell longevity. The analysis of the proliferation kinetics and persistence of MCMV-driven Ly49H^+^ NK cells showed that following a contraction phase, a long-lived and self-renewing memory cell pool persists for several months after infection in a variety of peripheral tissues, where it displays an enhanced response to secondary challenge [16]. The downregulation of the prosurvival molecule Bcl-2 and Bim-mediated proapoptotic signaling during the contraction phase regulate the size of the memory cell pool [53, 54]. Further, the survival of memory NK cells during the contraction phase after MCMV infection requires mitophagy of dysfunctional mitochondria, through an Atg3-dependent mechanism [55].

### 3.2. Resistance to Tumor-Dependent Immunosuppressive Microenvironment

Although NK cells are expected to target malignant cells and to play an important role in the immune surveillance against tumors, it is now appreciated that the suppressive components in the tumor microenvironment dampen the NK cell efficacy [56, 57]. Several studies have revealed a central role for Treg in suppressing tumor-infiltrating NK cells [58, 59]; in this context, Treg-mediated suppression of ADCC has been shown to correlate with a lower clinical efficacy of therapeutic tumor-targeting mAbs [60]. Treg can act both by secreting immunosuppressive cytokines (TGF*β*, IL-10, and IL-35) and by expressing inhibitory receptors (e.g., CTLA4 and PD-1) on their surface. Recent data uncover a new mechanism for Treg-mediated suppression of NK cells, based on the production of IL-37 which promotes the downregulation of the T cell immunoglobulin and mucin-domain containing-3 (Tim-3), that may behave as a stimulatory receptor in NK cells [61], and the upregulation of PD-1. Compared with cNK cells, whose proliferation, IFN-*γ* production, and cytotoxicity were efficiently inhibited by Treg, memory NK cells were found to be inherently resistant to Treg-mediated suppression, as they expressed low levels of IL-37 receptor, IL1R8, and PD-1, along with high levels of Tim-3 [62].

Further, NK cells express an inhibitory receptor called T cell immunoreceptor with immunoglobulin and immunoreceptor tyrosine-based inhibition motif domain (TIGIT), which also marks exhausted CD8^+^ tumor-infiltrating lymphocytes (TIL) [63]. TIGIT along with CD96 (also known as TACTILE) are coinhibitory receptors which by recognizing the same ligands of DNAM-1, namely, PVR (CD155) and nectin-2 [64, 65], counterbalance DNAM-1 activation at the NK-target synapse. Similar to T cells, *in vitro* blockade of TIGIT enhances cytokine secretion and cytotoxicity in NK cells [66–68] (Figure 1).

Recently, NK cell inhibition by myeloid-derived suppressor cells (MDSC) was shown to rely on TIGIT-PVR axis and was consequently abrogated upon TIGIT blockade. Also in this case, memory NK cells were found to be resistant to MDSC-mediated suppression in patients with cancer [68].

mAb-mediated interference with MHC class I-specific inhibitory receptors of NK cells can represent a strategy to potentiate their antitumor functions. In this regard, NKG2A blockade by means of a specific mAb (IPH2201, monalizumab) is currently being evaluated for a variety of tumor types in combination, for instance, with tumor-targeting mAbs [69]. The lack of NKG2A inhibitory receptor on memory NK cells makes such cells inherently resistant to HLA-E-expressing tumor-mediated inhibition and represents another advantage for the possible exploitation of these cells.

### 3.3. Amplified Functional Responses to IgG-Opsonized Targets

CD16 represents a prototype NK activating receptor; its engagement by IgG-opsonized targets is sufficient to trigger ADCC, as well as the production of proinflammatory cytokines and chemokines, such as IFN-*γ*, TNF*α*, IL-6, GM-CSF, and CCL5 [1, 2, 70, 71]. Human CD16 exhibits two extracellular Ig domains, a short cytoplasmic tail and a transmembrane domain that enables its association with ITAM-containing CD3*ζ* and Fc*ε*RI*γ* chains [72], which guarantee Syk- and ZAP-70-dependent signal transduction [2].

Notably, CD16-triggered ADCC and phagocytosis, performed by NK cells and macrophages, respectively, are among the main immune-dependent mechanisms by which tumor-targeting therapeutic mAbs mediate tumor cell killing [6–8].

A key feature of memory NK cells is their capability to mediate amplified Ab-dependent functional responses in terms of degranulation and cytokine production [19–21, 33, 34]. In particular, memory NK cells exhibit a greatly enhanced ability to produce IFN-*γ*, as a consequence of hypomethylated IFNG regulatory region [25], in response to activation via CD16, thus providing a prompt and powerful response against Ab-opsonized target cells. Indeed, despite the lower CD16 expression, they have been shown to more efficiently mediate polyfunctional responses, e.g., degranulation and IFN-*γ* production, upon stimulation via Ab-opsonized targets. The apparent conflict between higher CD16-triggered functional responses and lower surface receptor levels may be explained by the exclusive coupling of CD16 to CD3*ζ* chain in memory NK cells that, thanks to ITAM motif quantitative differences (3 ITAM in CD3*ζ* vs. 1 ITAM in Fc*ε*RI*γ*), may lead to more robust and efficient biochemical signals [70] (Figure 1). Moreover, the residual levels of CD3*ζ* chain may preserve the CD2/CD58 costimulatory interaction [73].

The enhanced response to CD16 stimulation has been well documented in response to antiviral IgG-opsonized infected cells and, of relevance here, to tumor-targeting therapeutic mAb-opsonized tumor cells [19, 21, 33, 34, 74]. Moreover, hyperresponsiveness to anti-CD20 mAb-opsonized tumor cells was also observed in *in vitro* expanded memory NK cells [74].

The capability of memory NK cells to activate in response to tumor cells has not been satisfactorily demonstrated yet. The reduction of NKp46 levels may explain the reduced ability of fresh and *in vitro* cultured memory NK cells to mediate effector functions in response to stimulation with K562 target cells, being its recognition largely dependent on this receptor [75]. However, NKG2C^+^ memory NK cells from HCMV-reactivating patients efficiently produced IFN-*γ* upon K562 stimulation [23, 44], indicating that the upregulation of other activating receptors may compensate for NKp46 defect. For example, CD2 ligand CD58, widely expressed by tumor B cells, has been shown to costimulate memory NK cell responses [33].

### 3.4. *In Vitro* Expansion Capability

*In vitro* expansion of NKG2C^+^ memory NK cells can be achieved by coculturing NK cells with CMV-infected fibroblasts or HLA-E-transfected cell lines [24, 76]. In these conditions, the interaction between CD94/NKG2C and its cellular ligand HLA-E, in combination with inflammatory cytokines, such as monocyte-derived IL-12, was critical for their expansion [77]. More recently, an HLA-E^+^ feeder cell-based protocol was shown to induce the selective *in vitro* expansion of memory NK cells that exhibited a profound skewing toward the expression of a single self-KIR, depending on the donor HLA-C genotype. These cells showed a high NKG2C-dependent cytotoxic potential against allogeneic pediatric acute lymphoblastic leukemia primary blasts [78], previously shown to be refractory to killing by allogeneic NK cells or NK92 NK cell line [79]. These data envisage a potential exploitation of memory NK cell alloreactivity in the context of novel adoptive cell therapy strategies.

Different lines of evidence highlight that primary HCMV infection drives the priming and proliferation of memory NK cells in a NKG2C-dependent manner [10–12, 76, 77]. HCMV-driven memory NK cell pool can be maintained by a variety of different viral super infections. In particular, an expanded population of memory NK cells was detected in EBV-, HBV-, HCV-, and HIV-seropositive individuals, only when patients were also seropositive for HCMV [18, 80]. It is therefore conceivable that Ab-mediated immune responses may drive the proliferation and maintenance of an already existing pool of memory NK cells, in some viral disease settings. Indeed, the capability of CD16-initiated signals to regulate NK cell proliferation and death, under selected conditions, has been shown [81, 82].

Seminal *in vitro* studies offered a mechanistic explanation for the role of virus-specific Abs in sustaining memory NK cell expansion and established a pivotal role for CD16 binding to antiviral IgG-opsonized cells to induce the proliferation of this specific subset [20, 21].

In this context, our recent data [74] demonstrate the unique capability of anti-CD20 therapeutic mAb-opsonized targets to drive the selective *in vitro* expansion of memory NK cells from HCMV-seropositive healthy donors. Indeed, we developed an effective *in vitro* culture system, consisting of a 9-day coculture of PBMC with irradiated lymphoblastoid Raji cells opsonized with anti-CD20 therapeutic mAbs, in IL-2-containing medium (Figure 1). Importantly, *in vitro* expanded memory NK cells, as their freshly isolated counterpart, displayed amplified CD16-polyfunctional responses upon stimulation with anti-CD20-opsonized tumor cells. It is conceivable that CD16-dependent memory NK cell proliferation also relies on multiple accessory signals, conveyed by cell-cell contacts and soluble mediators. In our system, ligands expressed by Raji lymphoblastoid B cells may provide accessory proliferative signals to memory NK cells; among them, CD2 ligand CD58, has been shown to costimulate memory NK cell responses [33]. Moreover, monocyte-derived IL-12, probably stimulated through Fc*γ*R engagement by anti-CD20-opsonized targets, likely mediates a critical contribution through the upregulation of CD25, as demonstrated by a recent report [77].

An extensive cell division, along with an induced expression of a functional high-affinity IL-2 receptor (IL-2R) *αβγ*, is also observed in cytokine-induced memory-like NK cells [39].

## 4. Perspectives

Based on their peculiarities, memory NK cell exploitment in adoptive therapy strategies is considered a particularly attractive tool in anticancer therapeutic perspective and is already a reality. Indeed, phase I clinical trials based on adoptive transfer of cytokine-induced memory-like NK cells for patients with relapsed or refractory AML [45], or *in vitro* expanded NK cells with an inducible adaptive phenotype in advanced cancer [83], are ongoing. The possibility to enhance memory NK cell antitumor functions through genetic manipulation has been suggested by a recent work showing that CAR-transduced terminally differentiated/adaptive NK cells exhibit superior effector functions when compared to other NK subsets [84].

Future studies are needed to uncover relevant aspects of memory NK cell biology in order to optimize their clinical application. A better definition of the phenotypic and functional heterogeneity in terms of tumor recognition capability, the possibility to *in vitro* manipulate or selectively expand memory NK cells endowed with selected receptor repertoire, the GMP-compliant adaptation of the procedure for their *in vitro* expansion will be instrumental for the better exploitment of NK cell memory for the ultimate benefit of treating cancer patients.

Importantly, the enhanced responsiveness and expansion capability in response to mAb-coated tumors may guide future attempts to combine strategies based on the adoptive transfer of *in vitro* expanded memory NK cells and tumor-targeting therapeutic mAbs, whose clinical responses are burdened by a significant proportion of relapses. Indeed, the promotion of an endogenous long-lasting adaptive antitumor immune response that may be highly relevant in maintaining long-term protection is becoming a major goal for improving the efficacy of mAb-based therapies.

It is worth investigating the possible contribution of memory NK cells to the development of the so-called “vaccinal effect” of therapeutic mAbs. Indeed, thanks to their amplified capability to produce cytokines upon mAb-opsonized tumor recognition, memory NK cells could participate to the development of adaptive antitumor immune responses, required for the long-term protection of mAb-treated patients [8, 85, 86].
